# Supplementary material for: Are carbon emissions trading and green financial instruments synergistic? -Comprehensive quantitative research based on content analysis
Source: PLoS One. 2024 Mar 7;19(3):e0298601. doi: 10.1371/journal.pone.0298601 (PMC10919601; doi:10.1371/journal.pone.0298601)
Supplement: S1 Data — (ZIP) [file pone.0298601.s001.zip › Data/地级市Z_F工作报告.zip!/░▓╗╒/╗┤▒▒╩╨/└┤╫╘╣1⁄2╓┌║┼í╛┬φ┐╦╩2╛▌═°í┐_2019.docx]

各位代表：

现在，我代表市人民政府，向大会报告政府工作，请予审议，并请各位政协委员和列席人员提出意见。

****一、2018年工作回顾****

2018年是极不平凡的一年。一年来，在省委、省政府及市委的坚强领导下，全市人民坚持以习近平新时代中国特色社会主义思想为指导，全面贯彻落实党的十九大和十九届二中、三中全会精神，坚定实施中国碳谷·绿金淮北战略，按照“一二三四五”总体发展思路，坚决扛起重大政治责任不松劲，坚持高质量转型发展不动摇，直面各种困难挑战不退缩，统筹推进稳增长、促改革、调结构、惠民生、防风险，经济社会发展取得新成效，多项重点工作实现新突破。预计，全年生产总值970亿元、增长4%，财政收入128.9亿元、增长19.8%，规模以上工业增加值与上年持平，固定资产投资增长9%，社会消费品零售总额324亿元、增长11%，城镇、农村常住居民人均可支配收入分别达到31944元、12598元，增长8%、8.5%。

****一年来，我们主要做了以下工作：****

****（一）精准服务实体经济，转型发展基础更加稳固****

始终把发展实体经济放在突出位置，出台降成本“新20条”，在降低企业制度性交易成本、人工成本、税费负担等方面打好组合拳。全年落实结构性减税20亿元、社保降费1.3亿元，发放稳岗补贴1.2亿元，安排产业扶持资金1亿元，节约企业用电用气成本2亿元。

深入开展“四送一服”活动和支持实体经济发展攻坚行动，积极走访困难企业和中小企业，对接要素项目4539个，办结企业反映问题1340个。针对融资难、融资贵突出问题，建立“金融超脑”服务平台，市农行在全省率先推出“万家e贷”普惠金融产品，徽商银行推出中标贷业务，多渠道解决中小企业融资需求。全市实现直接融资307.6亿元，累计为企业提供续贷过桥资金24亿元，发放“税融通”贷款6.6亿元，新增新型政银担合作贷款21亿元，利用商标质押等融资42亿元，44家企业挂牌省区域四板。出台促进民营经济加快发展“33条”，全年新登记私营企业7002户、个体工商户17726户，分别增长49.7%、9.2%。

为应对持续加大的经济下行压力，坚持把稳投资作为贯彻中央“六稳”要求的关键举措。强化项目带动，认真落实“四督四保”要求，加快在建项目建设进度，全年组织实施重点项目616个，完成投资459亿元。符夹线扩能工程建成通车，淮水北调市级配水工程建成通水。S101合相路二期、S254古毛路二期、S235濉唐路二期、G237济宁线、浍河南坪船闸航道整治一期工程等加速推进。

加快推进“僵尸企业”出清，全年处置“僵尸企业”60家，盘活土地4389亩。“三供一业” 分离移交工作基本完成。卧龙湖煤矿停产关闭通过省级验收，去产能工作得到省政府通报表扬。

****（二）着力推动转型升级，新旧动能转换更加强劲****

坚持创新第一动力，全面启动创新型城市创建工作，编制绿金科创大走廊产业发展规划。全市新认定高新技术企业26家，总数达到69家。濉溪铝基复合材料基地获批国家火炬特色基地。平山电厂135万千瓦机组成为全球燃煤发电绿色标杆。

聚焦“四基一高一大”战略性新兴产业，以“三重一创”为主抓手，制定战略性新兴产业三年发展规划。设立总规模25亿元的产业扶持基金，吸引社会资本参与设立陶铝新材料等4个子基金。烈山新型元器件获批省重大新兴产业工程。50万吨甲醇、50万吨苯乙烯项目完成前期工作，AG玻璃、甲艾马达等一批项目正式投产。陶铝研究院特铸生产线具备生产条件，中国商飞、中国铝业、上海交大高层来淮考察，与我市共同推进陶铝新材料产业化发展。实施新一轮传统产业技术改造升级工程，全年实施重点项目153个，技改投资增长10%。新增省级“专精特新”中小企业17家、安徽名牌产品7个、安徽工业精品7个。预计全市规模以上工业企业利税、利润总额分别增长10%、21%。口子东山产业园一期、中煤远大建筑产业化基地建成投产，华孚高档纺织面料项目开工建设。淮海实业集团跻身中国制造企业500强。

现代服务业发展步伐加快，中农批淮北大市场正式建成，隋唐运河古镇商业街投入运营。四季榴园、杭淮惠龙物流园获批省级服务业集聚示范园区。成功举办2018淮北食品工业博览会、葡萄采摘节、石榴文化旅游节。南湖景区跻身4A级。预计服务业占生产总值比重达38.1%，比上年提高1.6个百分点。

粮食生产实现“十五连丰”。粮食安全进一步巩固。临涣酱包瓜获评国家地理标志保护产品。非洲猪瘟疫情防控严密有效。实施农村电商全覆盖巩固提升行动，完成“两中心、一站点”建设任务，“快递下乡”工程实现全覆盖。濉溪县获批创建全国农村一二三产业融合发展先导区。淮北国家农业科技园区成功通过验收。

****（三）深化改革扩大开放，要素集聚效应更加显现****

“放管服”改革持续深化，动态调整市级政府权责清单，市级权力事项减少240项。扎实推进“一网一门一次”改革，在全省率先推行移动端全程电子化登记，实现“不见面”审批。开展“减证便民”专项行动，政务服务事项申请材料同比精简60.3%。深化“多证合一”改革，全面推开“证照分离”改革，企业开办时间压缩至3个工作日以内。公共资源交易全流程电子化招标率达到80%。社会信用体系建设不断加强。国税地税征管体制改革扎实推进。市建投集团成功定价发行全国首单城商行增信公募海外债，在开拓国际资本市场上取得新突破。

综合医改进展顺利，4家紧密型城市医联体挂牌运行，公立医院编制周转池制度全面推开。在全省率先建立公共卫生服务激励机制，激发公共卫生事业发展活力。农村“三变”改革积极推进，农村集体资产清产核资工作全面完成。国家中小城市综合改革试点成效明显，“房产超市”和“4321”融资担保金融服务入选全国经验案例。

对外开放不断扩大，预计全年实现进出口总额7.1亿美元，同比增长16.8%。淮北海关正式获批，为我市外向型经济发展注入新动力。开发区优化整合全面完成，全市获批省级开发区4个、省级高新区1个。招商引资取得新成效，全年新签约招商引资项目222个，新开工规模项目176个。理士新能源电池及电动车、万顺高精度铝箔生产线、盛美诺生物科技、东华软件、宁丰OSB板等一批优质项目落地淮北。

****（四）坚决打好三大攻坚战，全面小康步伐更加坚实****

开展涉嫌非法集资专项清理整顿，排查整治各类非法金融活动。强化预算执行和刚性约束，规范政府举债行为，加强隐患排查和风险防控，政府存量债务置换全面完成。

严格落实精准扶贫精准脱贫基本方略，坚持高位推动，层层发动，实施打赢脱贫攻坚战三年行动，持续推进“重精准、补短板、促攻坚”专项行动和四季攻势，夯实基层基础工作，落实产业扶贫到户项目1.7万个，开发公共服务岗位2500个，贫困人口家庭医生签约服务、建档立卡贫困学生资助实现全覆盖。切实抓好中央及省委巡视问题整改，提升脱贫质量，巩固脱贫成效，3个贫困村出列、3256人实现脱贫。

以中央环保督察问题整改和“回头看”活动为抓手，组织开展“大气污染防治百日攻坚”、固废专项检查与整治等行动，在大气、水、土壤污染防治方面打出组合拳。秸秆禁烧实现零火点，城区烟花爆竹禁燃成效明显，全年PM2.5平均浓度为58.4微克/立方米，同比下降11.9%。城市集中式饮用水水源水质达标率100%，县区126座粘土砖瓦窑厂全部关闭。烈山、杜集片区污水转输工程建成运营。旺能生活垃圾焚烧发电、生活垃圾卫生填埋场项目开工建设，城乡生活垃圾处理能力不足问题将得到彻底解决。我市入选全国建筑垃圾治理试点城市。

****（五）强力推进城乡统筹，城市建设发展更加均衡****

城市总体规划修编工作稳步推进，“城市双修”试点工作获住建部肯定，中心湖带及朔西湖片区规划编制基本完成。城市路网不断完善，桂苑东路、学院西路、方安南路等建成通车，泉山隧道全线贯通，泉山北路上跨立交桥、龙山北路加快建设。新增海绵城市10平方公里，建成地下综合管廊14.4公里，完成房屋征收263万平方米。

老城区功能进一步完善。滨河一期、热电厂小区等8个老旧小区完成改造，新建城市绿道38公里、公交候车亭16座、公厕6座，改造菜市场7家，新开通114路公交车，“十分钟便民生活圈”更加完善。“两治三改”工作有序开展，整治违法建筑14万平方米、违法用地1181亩。调整完善棚改政策，明确改造范围和界定标准，优化安置方式，完成棚户区改造35918套，群众居住条件进一步改善。无线城市基本建成，在全省率先实现“数字城管”市域一体化。我市荣获2018中国城市治理智慧化优秀奖。

实施农村人居环境整治三年行动，一体化推进农村环境“三大革命”，清理生活垃圾22万吨、完成改厕29735户。扎实推进美丽乡村建设，完成5个镇政府驻地建成区和28个中心村整治任务。出台奖补政策，支持县区加快“四好农村路”建设，完成农村公路畅通工程142公里、贫困村扩面延伸工程27.9公里，濉溪县实现城乡客运公交一体化，获评全省“四好农村路”建设示范县。全市9个镇级农村运输综合服务站、22个农网改造升级项目、农村饮水安全巩固提升工程、农田水利“最后一公里”工程和504户农村危房改造、450户因灾倒损房屋重修工程全面完成，群众得到更多实惠。“大棚房”清理取得初步成效。

持续推进生态文明建设，全面实行河（湖）长制、林长制。积极创建国家森林城市，完成林业增绿增效工程4.6万亩，三区荣获省级森林城市称号。老濉河水生态环境综合整治全面完成，呈现河畅、水清、岸绿、景美新面貌。节水型城市创建工作正式启动。在全省实行最严格水资源管理制度年度考核中，我市位居首位。以绿色发展理念创新开展采煤沉陷区综合治理，杜集区继濉溪县之后，获国家新一轮政策支持。

****（六）倾力办好民生实事，共建共享成果更加显著****

持续加大民生投入，财政用于民生领域支出达130.8亿元，民生工程继续保持全省领先。城镇新增就业5.2万人，城镇登记失业率控制在2.57%以内。扎实推进技工大市建设，新增技能人才2.8万人。实施第三期学前教育行动计划，新建、改扩建幼儿园8所。调整城区教育资源布局，维修改造老城区学校6所。新招聘教师545人。完善全面两孩配套措施，在全省率先开展3岁以下婴幼儿照护服务工作，着力解决家庭托育难题。大运河文化带建设积极推进。我市健儿在第十四届省运动会、第七届省残运会取得好成绩。积极应对人口老龄化挑战，我市成功入选全国第三批居家和社区养老服务改革试点。

农村低保标准大幅提升，企业退休人员基本养老金实现“十四连涨”。成功创建省级食品药品安全城市。安全生产“铸安”行动常态化和“六项机制”规范化不断深入，“深督导、重化解、促落实”专项行动扎实开展，扫黑除恶专项斗争成效显著，破获20年前命案积案5起，群众安全感和满意度保持全省领先。加大房地产领域历史遗留问题攻坚力度，南黎花园等3个试点顺利完成，为全面解决“办证难”积累了经验。烈山区荣获全国“法治县（区）创建活动先进单位”，杜集区双楼村被评为全国“民主法治示范村”。村（居）换届工作全面完成。“一组一会”成为基层治理新亮点。

文明创建持续深化，市容环境治理、礼让斑马线、文明志愿服务深入开展，“中国好人”总数达149位，人口占比稳居全国第一。我市再获“全国无偿献血先进市”称号。第四次全国经济普查取得阶段性成果。工会、老年人、妇女儿童、青少年、残疾人、红十字等事业持续推进，民族宗教、外事侨务、对台等工作不断加强，档案、地方志、保密、气象、供销、机关事务、应急管理等服务发展能力进一步增强。

积极支持国防和军队改革，国防教育、国防动员、人民防空、退役军人服务管理和优抚安置工作取得新成绩。

****（七）持续提升履职能力，忠诚干净担当更加有为****

认真贯彻新时代党的建设总要求，扎实开展“讲忠诚、严纪律、立政德、善担当、新作为”专题警示教育，认真完成省委巡视反馈涉及政府系统12个问题整改任务。大力推进法治政府建设，新修订市政府工作规则，完成新一届市政府法律顾问聘任，邀请市民代表列席市政府常务会议，《淮北市养犬管理办法》《淮北市燃放烟花爆竹管理办法》正式实施。认真执行市人大及其常委会决议、决定，主动接受市政协民主监督，办理人大代表议案、建议129件，政协委员提案184件。政府网站集约化云平台开通上线，各类资源实现优化整合。全市统一的“12345”政府服务热线平台正式运行，为群众解决实际问题1.3万件。严格落实中央八项规定精神，坚决反对形式主义、官僚主义，深入推进党风廉政建设和反腐败斗争，加强目标管理和政务督查，强化审计监督，营造了干净干事、风清气正的良好环境。

一年来，我们在统筹推进各项工作的同时，聚焦“四转一享” 主攻方向，坚持抓主抓重，持续发力，取得一批标志性成果。

一是在国务院第五次大督查中，淮北市积极扶植新兴产业、促进资源枯竭型城市转型的典型经验做法，先后获国务院办公厅、省政府办公厅通报表扬。

二是财政收入增幅全省第一，总量和增幅创历史最好水平。

三是陶铝新材料列入工信部重点新材料首批次应用示范指导目录，军民融合发展迈出坚实步伐。

四是淮北矿业成功实现重组上市，为打造更具生机活力的大型现代能源化工集团奠定了坚实基础。

五是新型煤化工基地十大碳基新材料项目开工建设，千亿园区产业集聚效应加速释放。

六是万达广场开业运营，新城吾悦广场全面建设，城市商业业态明显提升。

七是老城区雨污分流工程全面启动，在城市黑臭水体治理方面率先打好标志性战役。

八是绿金湖治理基本完成，城市“拥湖发展”的空间格局初步形成。

九是采煤沉陷区治理获国家发改委、自然资源部充分肯定，我市入围第十届中华环境奖。

十是成功战胜特大暴雨灾害，全市没有出现一例人员伤亡，最大限度保障了人民群众生命财产安全。

回顾一年来的工作，我们深刻体会到，进入新时代，只有坚持以人民为中心，不断满足人民日益增长的美好生活需要，才能得到全市人民的理解和支持，凝聚各方面的智慧和力量，战胜各种困难、风险和挑战。这些成绩必将同全市人民长期奋斗的成就一起，成为我们推动高质量发展、加快转型崛起的基础所在、信心所在、希望所在。这些成绩离不开省委、省政府及市委的坚强领导，离不开市人大、市政协的监督支持，是全市人民团结奋斗的结果。在此，我代表市人民政府，向各位人大代表、政协委员，向各民主党派、工商联、无党派人士和人民团体，向驻淮部队、武警官兵、政法公安干警和消防救援队伍指战员，向离退休老同志和关心、支持、参与淮北改革发展的企业家和各界朋友，表示衷心的感谢！

回顾一年来的工作，我们也清醒看到，我市经济下行压力较大，生产总值、规模以上工业增加值与年初计划差距较大，中小企业面临困难较多，重大工业项目招引不足，经济增长的基础还不稳固。创新驱动能力不强，民营经济发展不充分。生态环境保护任务繁重，大气污染防治异常艰巨。民生领域尤其是农村基础设施和公共服务方面仍有不少短板，全市还有3000多人尚未脱贫。少数干部创新能力不足、担当精神不够，官僚主义、形式主义时有发生。对此，我们将始终坚持问题导向，高度重视并认真加以解决，决不辜负全市人民的期盼。

****二、2019年重点工作****

今年是新中国成立70周年，是决胜全面建成小康社会的关键之年。综合分析，尽管我们面临经济下行压力加大、区域竞争激烈等多种挑战，面临提升经济实力、转换增长动力等多重考验，不确定的因素在增加，但可以确定的发展机遇也很多。从外部环境看，我国发展仍处于并将长期处于重要战略机遇期，而且这个重要战略机遇期具有新的内涵，同时，中央继续实施积极的财政政策和稳健的货币政策，“六稳”政策效应逐步释放，省委、省政府支持资源型城市加快转型，我市纳入淮河生态经济带发展规划，淮海经济区核心区城市交流合作日益深化。从自身发展看，经过长期的实践探索，我市已经积累了应对困难的丰富经验，积蓄了加快发展的坚实后劲，积淀了伟大的创城精神和抗洪精神，积聚了转型崛起的强大力量。只要我们坚定信心决心，保持定力韧劲，勇于担当作为，就一定能成功跨越一个又一个关口，实现更高质量、更可持续的发展，推动各项工作奋力赶超、争先进位。

今年政府工作的总体要求是：以习近平新时代中国特色社会主义思想为指导，全面贯彻党的十九大精神和中央、省委经济工作会议精神，按照市委八届八次全会部署，坚持稳中求进、进中求好工作总基调，坚持新发展理念，坚持推动高质量发展，坚持以“巩固、增强、提升、畅通”八字方针推进供给侧结构性改革，坚持深化市场化改革、扩大高水平开放，坚定实施中国碳谷·绿金淮北战略和“一二三四五”总体发展思路，统筹做好“六稳”工作，保持经济持续健康发展和社会大局稳定，为全面建成小康社会收官打下决定性基础，以优异成绩庆祝新中国成立70周年。

经济社会发展的主要预期目标是：全市生产总值增长6%以上，规模以上工业增加值增长6%，固定资产投资增长8%，财政收入增长8%以上，进出口总额增长11%，社会消费品零售总额增长10%，城镇、农村常住居民人均可支配收入分别增长8%、8.5%，城镇调查失业率5.5%以内，节能减排完成省定目标任务。

贯彻总体要求，实现预期目标，需要深刻把握淮北转型发展的阶段性特征，深刻洞察高质量发展的战略方向，深刻体悟人民对美好生活的向往，坚持问题导向和目标导向相统一，整体推进与重点突破相结合，即期增长与长远发展相促进，抓重点、补短板、强弱项，一件事接着一件事办，一年接着一年干，力争每年都有一批标志性成果，更好地解决发展中的不充分和不平衡问题。

今年重点推进十大工程：市科创中心、100万吨碳基新材料、陶铝航材基地、平山电厂二期、铝箔系列项目、完美生物科技、市高新区50万平方米标准化厂房、老城区雨污分流、旺能生活垃圾焚烧发电、淮宿蚌城际铁路。

****着力抓好八个方面的工作：****

****（一）坚持稳定增长与转型升级同向发力，推动经济高质量发展。坚持工业立市、产业兴市理念，在全力以赴稳增长的同时，持续扩大有效投入，积极构建现代产业体系。****

****一是推动实体经济健康发展。****全面落实降低企业成本各项政策，在降低企业合规成本和要素成本上协同发力，进一步精简行政审批事项，严格规范涉企收费，实质性减轻企业负担。常态化开展“四送一服”活动，精准对接企业需求，建立问题清单和任务清单，着力解决企业实际困难。优化金融供给，积极搭建政银企合作平台，引导银行机构加大信贷投入。创新金融服务，发展普惠金融，扩大企业抵质押物范围，降低小微企业融资成本。持续扩大电力直接交易规模，让更多企业享受电价优惠。深化人才招聘帮办活动，组织实施“春风行动”，缓解企业“招工难”。

****二是构建现代产业体系。****统筹推进制造强市和质量强市建设，不断提高发展质量和效益。培育壮大新兴产业，围绕“四基一高一大”战略性新兴产业，强力推进“三重一创”建设，争取一批重点项目纳入省级政策支持范围。完善“基地+基金”模式，探索设立股权投资基金，加大对新兴产业和优质企业支持力度。全面深化政产学研用合作，加快铝基高端金属材料产业研发和质量检验平台建设。与中国商飞、中国铝业、上海交大协同推进陶铝航空新材料生产基地建设，合力打造“新一代航空材料”，争创国家军民融合产业示范基地。改造提升传统产业，全年实施产业升级和技术改造重点项目140个，技改投资增长9%。继续开展质量提升行动，围绕增品种、提品质、创品牌，筛选培育一批省级制造业创新中心、技术创新示范企业，新增省级企业技术中心4个、安徽工业精品5个、新产品15个。深化“互联网+先进制造业”，启动“企业上云”计划，创建数字化车间4个。做好“小升规”企业培育，新增规模以上工业企业60家。加快发展现代服务业，新城吾悦广场、中农批淮北大市场投入运营，推动商贸服务业由带状分布向现代化商圈升级，促进现代物流、金融、科技服务等生产性服务业集聚发展，繁荣城市经济。建立和完善电子商务市场体系，争创一批国家级或省级电子商务示范园、跨境电商示范园。积极做好中心湖带文化旅游产业布局，扎实推进相山区、烈山区全域旅游示范区创建工作，规划建设东部沿山生态旅游示范带，继续办好淮北食品工业博览会、葡萄采摘节、石榴文化旅游节等活动。着力培育智慧经济，推进数据资源整合共享和有序开放，强化与科大讯飞、东华软件等企业战略合作，开展数据资产变现与增值业务，创新发展大数据、云计算、人工智能等数字产业。

****三是推进重大项目建设。****发挥政府性投资的引导作用，鼓励社会投资，集中向新型工业化重要领域、向县区和园区重大项目、向基础设施重点工程倾斜，不断增强发展后劲。全年新建、续建和储备重点项目615个以上，完成投资465亿元以上。推动50万吨甲醇、50万吨苯乙烯、华孚绿尚小镇等项目开工，平山电厂二期基本建成，英科医疗、完美生物科技、中冶淮海、康美绿筑等项目正式投产。S101二期、S202改扩建等骨干道路建成使用。抢抓国家推动新一轮稳投资政策机遇，力争淮宿蚌城际铁路尽快开工建设，积极推进淮徐快速通道、徐淮阜高速公路、浍河航道二期、港口码头及临涣船闸等重大项目。在采煤沉陷区综合治理、老工业区搬迁改造和资源枯竭城市转型等重点领域，继续加大政策和项目争取力度。

****（二）坚持招商带动与创新驱动并进联动，壮大转型崛起新动能。牢固树立创新第一动力、招商第一要事、人才第一资源、环境第一保障的意识，千方百计扩大增量、优化存量、提升质量，加快新旧动能转换。****

****一是强力推进招商引资。****坚持把招商引资作为经济工作的头号工程，坚定不移推进精准招商、产业招商和以商招商，加强项目质量评估，提高招商质量和效益。紧盯“四基一高一大”战略性新兴产业，着力招大引强，加快引进一批上下游关联企业，推动产业集聚。依托产业基地、工业园区，发挥产业引导基金、股权投资引导基金作用，积极推进“基金+招商”“基地+招商”，创新产融结合招商新模式。进一步加强招商队伍建设，不断提高专业化招商水平。顺应产业和技术发展趋势，加大招才引智力度，健全人才柔性流动机制，积极招引高端人才和科研院所，全年引进高层次科技人才团队5个以上、高层次科技人才100名以上，以人才链补强创新链、拉长产业链。落实各项激励政策，强化科技研发、创业融资等服务配套，完善住房、教育等生活保障，让各类人才在淮北愉快创业、愉快生活、实现梦想。

****二是扎实推进创新型城市建设。****按照“一年打基础、三年见成效、五年上台阶”目标，协同推进科创大走廊、东部科教新城、国家高新区“三区联创”，开工建设市科创中心。积极参与长三角创新城市联盟交流合作，主动对接省“四个一”创新主平台，争取将上海交大（安徽）淮北陶铝研究院列入合肥综合性国家科学中心分中心，加快构建协同创新体系，提升创新能力和效率。深入推动与中科院、清华大学、煤科总院、华东师范大学等大院大所合作，加强淮北师范大学、淮北职业技术学院服务地方能力建设，举办好碳基新材料、铝箔、芳香产业高峰论坛或年会，促进科技研发和成果转化。加快中科（淮北）产业技术研究院建设，力争智能制造工业互联网研究中心、云计算大数据中心和物联网传感器研究中心建成运行。加强源创客科技孵化基地等平台建设，打造大众创业、万众创新升级版。

****三是着力打造“四最”营商环境。****以“放管服”改革为抓手，进一步深化“互联网+政务服务”，推进“一网一门一次”改革，深化“证照分离”改革，大力推进网上审批，优化办事流程，提高办事效率，让企业和群众办事像网购一样方便。启动新政务服务中心建设。构建亲清新型政商关系，大力激发和弘扬企业家精神，主动当好服务企业的“店小二”，做到有事必到、无事不扰，建立法治化营商环境，进一步增强微观主体活力，更好发挥企业和企业家主观能动性。

****（三）坚持深化改革与扩大开放深度融合，打造内陆开放新高地。以改革开放40周年为新起点，全面推进各项改革，主动融入国家及省发展战略，在更高水平、更高层次上推动新一轮改革开放。****

****一是不断深化重点改革。****全面深化“双随机一公开”监管，实现市场监管全覆盖。深化国资国企改革，健全国有资本经营预算管理制度，逐步提高国有资本收益上缴公共财政比例，促进国有资产保值增值。支持市建投集团以市场化经营推动改革创新，优化资产配置，做强核心业务，提高综合竞争力。深入推进财政预算管理改革，加快建立全方位、全过程、全覆盖的预算绩效管理体系，推动财政事权与支出责任划分改革，做到花钱必问效、无效必问责。深化金融改革，推进企业上市挂牌，扩大直接融资规模。深化公共资源交易监管体制改革，提高公共资源配置效率和效益。深化农村综合改革。推进农村土地“三权”分置，促进农业规模化经营。全面完成农村集体产权制度改革，实现涉改村（居）全覆盖，进一步壮大村集体经济。

****二是持续扩大对外开放。****深度对接国家“三大战略”，积极融入淮河生态经济带、淮海经济区核心区，加强与省际省内城市交流互动，精心组织淮海经济区协同发展座谈会。大力推进外贸增长工程，加大对龙头企业扶持力度，孵化培育外贸中小企业。充分发挥口岸服务优势，推进淮北海关挂牌、青龙山铁路海关监管中心运营，降低外贸企业物流成本，提高进出口便利化水平。积极参加中国国际徽商大会、上海国际进口博览会，组织更多企业“请进来”“走出去”。

****三是发展壮大民营经济。****民营企业和民营企业家是我们的自己人。全面落实促进民营经济发展各项政策，营造公平竞争的市场环境，消除各种隐形壁垒和歧视，促进国资、民资、外资一视同仁、共同发展。依法保护民营企业合法权益，执行政策不搞“一刀切”，厚植民营企业家成长沃土。全年新登记私营企业5000户、个体工商户1.2万户，民营经济增加值占生产总值比重提高1个百分点。动态完善企业分类培育库，推动20家龙头企业、100家骨干企业晋档升级。聚焦中小企业“专精特新”，培育“单项冠军”和“隐形冠军”，新增省级“专精特新”企业15家。建立服务民营企业常态长效机制，在各省级开发区设立服务企业办公室，让政府和民营企业沟通更直接。

****（四）坚持品质提升与功能完善相互统筹，提高城乡发展协调性。围绕全域城镇化目标，坚持规划引领，优化布局，促进老城区有机更新，新城区产城融合，推动城乡一体、协同发展。****

****一是完善规划体系。****积极推进城市总体规划修编报批，完善市县空间规划及数据库，探索建立“多规合一”空间规划体系，优化城市空间布局。组织编制城市建筑风格和色彩规划、城市高层建筑布局等专项规划，加强对重点片区建设与更新的规划指引。完善“城市双修”专项规划，总结经验亮点，做好国家试点验收准备工作。

****二是提升老城区功能品质。****加快古城东路、开渠西路、渠沟东路铁路立交桥等续建项目建设，开工建设环山东路、机厂路改造工程，建成泉山北路上跨立交桥及辅道工程，着力破解交通拥堵难题。深入开展“两治三改”三年专项行动，有序推进棚户区改造，依法依规控制棚改成本，基本建成8904套。推进老旧小区“微更新”，改造老旧小区5个。积极开展既有住宅加装电梯试点，提升居民居住品质。围绕办好群众家门口的事情，构建“十分钟便民生活圈”，新建或改造一批菜市场、公厕、游园节点。扎实推进新型智慧城市建设，增强城市治理数字化应用能力。加强海绵城市建设，完善基础设施，提高综合抗灾减灾能力，打造安全韧性城市。

****三是加快新城新区建设。****明晰城市发展定位，完善配套支持政策，统筹推进东部新城、南部次中心、高铁新区、凤凰新区、双堆集中心镇新城建设，促进市县统筹、城乡一体。创新土地增减挂钩机制，推进农村建设用地复垦建新，盘活闲置低效土地，保障城乡建设用地需求。加快东部新城建设，建成泉山隧道、东部新城路网，市一中新校区、人民医院东院区主体工程完工。支持县区完善基础设施和城市功能，积极推进人民西路西延、西山北路、朔西湖环湖步道、杨河北路、湖西路和花山公园建设，启动濉溪天然气供气专线及配套场站建设。支持烈山教育小镇建设。

****（五）坚持补齐短板与挖掘潜力有机结合，坚决打好三大攻坚战。把打好三大攻坚战作为全面建成小康社会的关键举措，精准聚焦，稳扎稳打，持续攻坚，努力把短板变成“潜力板”。****

****一是坚决打好防范化解重大风险攻坚战。****压实风险防范主体责任，强化政府预算管理和债务限额管理，进一步优化财政支出结构。规范政府举债融资机制，加强债务风险防控，减少存量、严控增量。加强政府投资项目管理，明确资金来源，注重投资效益分析，坚决杜绝盲目举债搞建设。加强金融监管，规范融资行为，深入推进非法集资、各类交易场所和互联网金融风险专项整治，筑牢金融风险防火墙。

****二是坚决打好脱贫攻坚战。****按照三年行动计划要求，加大精准扶贫力度，确保2000左右贫困人口实现脱贫。把临涣镇、韩村镇、双堆集镇革命老区脱贫攻坚摆在更加突出的位置，坚持精准定位、精准布局、精准落实，重点解决“两不愁三保障”。加大产业就业扶贫力度，实施产业扶贫项目提升、“四带一自”深化、“一村一品”推进三大行动，拓展“三业一岗”就业扶贫，建设一批就业扶贫驿站。大力实施扶贫扶智工程。统筹推进贫困村与非贫困村脱贫攻坚，健全防范返贫机制，解决“边缘户”实际困难，确保脱真贫、真脱贫。强化“单位包村、干部包户、全员参与”工作机制，调动全社会力量参与，构建大扶贫格局。

****三是坚决打好污染防治攻坚战。****持续抓好中央环保督察突出问题整改，巩固整改成效，在解决突出环境问题上持续发力。继续聚焦“三大保卫战”和“七大标志性战役”，进一步加大工作和投入力度，确保全市生态环境质量不断改善。加快推进老城区雨污分流工程，加大城市黑臭水体治理力度，还群众水清岸绿、鱼翔浅底美丽景象。强化土壤污染管控和修复，统筹抓好生活垃圾分类、餐厨垃圾无害化处置、建筑垃圾资源化利用等改革试点，旺能生活垃圾焚烧发电项目建成运营，生活垃圾卫生填埋场投入使用，提升城乡生活垃圾综合处理能力。鼓励绿色产品消费升级，倡导绿色出行，落实公交优先发展政策，更新置换新能源公交车200台。

完善环境保护制度体系，全面落实河（湖）长制、林长制，严格“三条红线”“四项制度”。加强生态环境保护和修复工作，扎实推进国家森林城市、节水型城市创建工作。高标准实施绿金湖景观绿化工程，高规格做好全国采煤塌陷地生态修复现场会各项准备工作，全面展示淮北创新之美、生态之美、发展之美，打造全国生态文明建设样板。

****（六）坚持乡村振兴与县区经济双向突破，提高县区综合竞争力。完善政策体系，加大支持力度，增强发展联动性，推动县区和园区在全省位次整体提升。****

****一是加快乡村振兴步伐。****制定乡村振兴扶持政策，整合资源力量，持续深入推进。以改善农村人居环境为突破口，深入推进农村环境“三大革命”，全年完成改厕23307户，农村生活垃圾无害化处理率达到75%以上，乡镇政府驻地污水处理设施全面建成。完善农村基础设施，完成“四好农村路”扩面延伸工程398公里，继续推进2018年度27个美丽乡村中心村建设，启动实施2019年度30个中心村规划建设。深化农业供给侧结构性改革，促进百善、凤凰山、高岳、榴园等农业示范园区转型升级，新增农业产业化联合体15个，创建优质农产品公共品牌，进一步落实粮食安全责任制。推进农村电商全覆盖巩固提升，大力发展休闲农业和乡村旅游，促进一二三产业融合发展。深入抓好“大棚房”专项清理整治行动，推动问题全面整改、彻底整改。扎实做好非洲猪瘟疫情防控，确保在我市不侵入、不扩散。针对洪涝灾害暴露出的薄弱环节，推进河道整治、河湖连通和涵闸除险加固，提高河道防洪除涝和水资源利用能力。

****二是加快县区经济发展。****坚持新型工业化、新型城镇化和农业现代化互动融合，立足县区实际，聚焦错位协同，利用优势资源发展优势产业，充分激发县区经济活力。推动县域产业集聚与特色镇域产业集群发展，争创全省首批县域特色产业集群（基地）。加快推进濉溪县、杜集区国家重点采煤沉陷区综合治理工程试点建设。稳步推进相山区、烈山区老工业区搬迁改造。加快石榴小镇、芳香小镇等特色小镇建设。支持农民工、高校毕业生和退役军人等各类人员返乡下乡创业，搭建创业平台，优化创业环境，推动更多人才、技术、资本等发展要素向县区汇聚。

****三是推动园区争先进位。****坚持高质量发展方向，落实各项支持政策，健全开发区考核机制，激发园区发展活力。深度对接园区需求，发挥市建投集团融资平台优势，通过政府引导、市场运作、合理回报等方式，建设类型多样的标准化厂房、孵化器、加速器，实现多方共赢。坚持以“亩均论英雄”，严把安全、环保、用能等标准，提升土地投资强度和产出率。基本解决“僵尸企业”问题，盘活存量资源，加快“腾笼换鸟”。加强园区管理体制改革，推广“管委会+公司”模式，提高市场化运营和管理水平。积极推动淮北高新区以升促建争创国家级高新区、濉溪开发区争创国家级经开区，支持新型煤化工基地打造现代化千亿产业园区。

****（七）坚持保障民生与促进和谐高度统一，提升人民群众幸福感。突出民生为先，扎实推进省市民生工程，增加公共服务供给，着力解决好群众关心关注的热点难点问题，让群众感受到更有温度的发展。****

****一是切实强化民生保障。****坚持就业优先战略，开展重点群体就业援助活动，推广小老板培育工程，以创业带动就业，新增城镇就业4万人。深入实施全民参保计划，推动“三保”融合，推进基本医保全国联网和异地结算工作。继续提高低保、特困供养等社会救助标准。深入开展全国居家和社区养老服务改革试点，实现城市居家养老服务三级中心全覆盖。大力推动医养结合养老产业发展，加快朔西湖健康小镇规划建设。积极促进和加强3岁以下婴幼儿照护服务，坚持边试点、边总结、边推广，不断完善家庭发展政策。完善农民工工资清欠机制，保障农民工劳动报酬权益。

****二是繁荣发展社会事业。****加快居民小区配套教育设施移交工作，补齐学前教育短板，加强乡村小规模学校建设，实施新一轮义务教育学校标准化建设和全面改薄工程。创新职业教育发展和职业技能培训，深化产教融合、校企合作。支持社会力量办学，促进民办教育发展。全面深化新时代教师队伍建设改革，建立中小学编制周转池，加强师德师风建设，完善待遇保障机制。巩固拓展医改成果，完善紧密型城市医联体建设配套措施，深入推进分级诊疗，加强基层医疗卫生服务体系和全科医生队伍建设，提高家庭医生签约服务水平。建成市紧急医疗救援指挥中心。认真做好省级卫生城市评估验收，同步启动国家卫生城市创建工作。加强大运河等历史文化遗产保护和传承利用，提升公共文化和体育产品供给能力。提高全民科学素质。认真做好第四次全国经济普查工作，做实数据，摸清家底。巩固全国文明城市创建成果，研究出台《文明行为促进条例》，推动文明创建常态化、长效化，厚植城市精神。

****三是不断创新社会治理。****扎实推动法治淮北、平安淮北建设，加强重点对象和社会普法，完善立体化信息化社会治安防控体系，深化扫黑除恶专项斗争。强化食品药品安全监管，巩固全省食品药品安全城市创建成果。坚持发展新时代“枫桥经验”，构建多元化解纠纷促进机制，依靠群众就地化解矛盾。严格落实安全生产责任制，深化安全监管体制改革，提升城市安全水平。健全应急管理机制，全面提升防灾减灾救灾能力。

全力支持国防和军队改革，积极做好退役军人服务管理工作，推进军民融合深度发展，进一步巩固全国双拥模范城创建成果。

****（八）坚持履职能力与工作质量同步提升，全面加强政府自身建设。民之所望，政之所向。我们要牢记使命担当，全面增强履职能力，不断提高政府工作质量，建设人民满意政府。****

****一是对党忠诚、牢记宗旨，不断提高领导力。****做好政府工作必须坚持和加强党的全面领导，永葆对党绝对忠诚的政治本色。树牢“四个意识”，坚定“两个维护”，认真开展“不忘初心，牢记使命”主题教育，把全面从严治党各项要求落到实处。始终把实现人民对美好生活的向往作为奋斗方向，加强学习和调查研究，培养专业素养、专业精神，创造性贯彻落实中央各项方针政策及省市决策部署，让对党忠诚、为民造福成为政府最鲜明的政治担当。

****二是马上就干、真抓实干，不断提高执行力。****推动淮北转型崛起，关键靠苦干实干马上干。坚持干字当头，实字为本，大力弘扬汗摔八瓣、皮掉几层的顽强作风，敢扛事、愿做事、能干事，确保政府各项工作高效运转、高质量推进。咬定目标不放松，奔着问题去，迎着困难上，敢闯敢干，踏实肯干，以钉钉子精神抓落实，确保工作成果经得起实践、人民和历史的检验。

****三是密切协同、优质高效，不断提高创新力。****全面落实政府机构改革各项任务，理顺职责关系，突出优化协同高效，确保上下贯通、左右协调、运行顺畅。坚持发展为要，事业至上，用好考核指挥棒，完善正向激励政策，激励政府系统全体人员新时代新担当新作为。坚持以上率下，当好实干家，以担当带动担当，以作为促进作为，提振干事创业精气神，释放创新创造新活力。

****四是依法行政、坚守底线，不断提高公信力。****严格按照政府工作规则依法行使权力，以制度刚性约束权力任性。全面推进政务公开，基本完成法治政府建设。自觉接受人大法律监督、工作监督和政协民主监督，广泛接受社会和舆论监督，让权力在阳光下运行。始终把纪律和规矩挺在前面，严格落实党风廉政建设主体责任和“一岗双责”，加强巡视巡察成果运用。牢固树立过紧日子的思想，严格压缩一般性支出，加大对重点领域的支持力度。深入贯彻中央八项规定精神，坚定不移纠“四风”、树新风，坚决反对“痕迹管理”“文山会海”等官僚主义、形式主义。加强政务督查和审计监督，严查发生在群众身边的腐败问题。

在新时代推动淮北转型崛起的前进道路上，风雨兼程方显本色，风雨无阻才见担当。让我们在中共淮北市委的坚强领导下，以永不懈怠的精神状态和一往无前的奋斗姿态，迎难而上，破难前行，不断实现人民对美好生活的向往，奋力开创中国碳谷·绿金淮北建设新局面，以优异成绩庆祝中华人民共和国成立70周年！
